# Spatial confinement is a major determinant of the folding landscape of human chromosomes

**DOI:** 10.1093/nar/gku462

**Published:** 2014-07-02

**Authors:** Gamze Gürsoy, Yun Xu, Amy L. Kenter, Jie Liang

**Affiliations:** 1 Department of Bioengineering, University of Illinois at Chicago, Chicago, IL 60607, USA; 2 Department of Microbiology and Immunology, University of Illinois College of Medicine, Chicago, IL 60612, USA

## Abstract

The global architecture of the cell nucleus and the spatial organization of chromatin play important roles in gene expression and nuclear function. Single-cell imaging and chromosome conformation capture-based techniques provide a wealth of information on the spatial organization of chromosomes. However, a mechanistic model that can account for all observed scaling behaviors governing long-range chromatin interactions is missing. Here we describe a model called *constrained self-avoiding chromatin* (C-SAC) for studying spatial structures of chromosomes, as the available space is a key determinant of chromosome folding. We studied large ensembles of model chromatin chains with appropriate fiber diameter, persistence length and excluded volume under spatial confinement. We show that the equilibrium ensemble of randomly folded chromosomes in the confined nuclear volume gives rise to the experimentally observed higher-order architecture of human chromosomes, including average scaling properties of mean-square spatial distance, end-to-end distance, contact probability and their chromosome-to-chromosome variabilities. Our results indicate that the overall structure of a human chromosome is dictated by the spatial confinement of the nuclear space, which may undergo significant tissue- and developmental stage-specific size changes.

## INTRODUCTION

Human cells must accommodate ∼6 billion base pairs of deoxyribonucleic acid (DNA) in a small nucleus with a diameter of 6–20 μm (1). Understanding the spatial organization of chromatin within the cell nucleus is key to gaining insights into the mechanism of gene activities, nuclear functions and maintenance of cellular epigenetic states (2). A major task is to understand the rules that govern the regulation of long-range chromatin interactions (2–4).

Fluorescence *in situ* hybridization (FISH) and chromosome conformation capture (3C) and related techniques revealed a wealth of information about spatial chromatin structures across different genomic regions for a variety of cell types (3,5–10). A key outcome of FISH experiments is the relationship between the mean-square spatial distance, *R*^2^, and the genomic distance, *s*, of two chromosome loci (5–6,9). The folded structures of chromatin fibers follow a scaling relationship of *R*^2^(*s*) ∼ *s*^2*ν*^. In human Chr 1 and 11, the exponent *ν* is ∼0.33 at smaller genomic (0.4–2 Mbp) distances, but levels off (*ν* ∼ 0) at larger genomic distances (>10 Mbp) (6). In mouse Chr 12, *ν* is found to be ∼0.25 and ∼0.37 for two different cell types at smaller genomic distances (<0.5 Mbp), and levels off at larger distances (>0.5 Mbp) (9). The leveling-off effects indicate that each chromosome is confined to a volume much smaller than the nuclear volume (6,9). This reflects the requirement that chromosomes must fit into localized territories (11).

Results from genome-wide 3C (Hi-C) experiments showed that the contact probability *P*_c_(*s*) between loci separated by a genomic distance *s* follows a power law of *P*_c_(*s*) ∼ 1/*s^α^*. The exponent *α* is ∼1.08 at genomic distances between 0.5 and 7 Mbp, when averaged across all chromosomes in a human cell line (3). Further analyses showed that chromosomes 11 and 12 exhibit the average human genome scaling behavior, with an exponent *α* ∼ 1.08 (3,12–17) while exponents for chromosomes X and 19 deviate from the average values significantly, with *α* ∼ 0.93 and ∼1.30, respectively (12). Similar results were obtained from a different Hi-C study (18) (see also (12) for analyses).

In order to gain understanding of the principles of spatial organization of chromatin, several polymer models have been developed (3,12–17,19–21). The fractal globule (FG) model (3,16) offers an explanation of the scaling of *P*_c_(*s*) and *R*^2^(*s*) with *s* at short genomic distances, although it does not account for the leveling-off effects observed in FISH studies (6,9). The FG model also does not explain the observed variation in α among different chromosomes. By attaching diffusible binders to chromatin, the Strings and Binders Switch (SBS) model can account for both the leveling-off effects and the heterogeneous scaling of α (12). However, individual scaling properties can exist only under carefully tuned conditions of binder concentrations and binding site distributions, which are unknown *a priori*. In addition, the SBS model does not exhibit multiple scaling exponents occurring simultaneously under one set of conditions.

The most important factor that determines how chromosomes fold in the cell nucleus is the amount of available space. In a recent study, spatial constraints were shown to be sufficient to produce the overall structural architecture of budding yeast genome (22), although the general effects of spatial confinement on chromatin folding of human genome are unknown. Polymer models can provide important insights into chromatin compaction in cell nucleus (19–21). However, a major obstacle in studying chromatin fibers confined in a small volume is the difficulty in generating a large number of unbiased model chromatin fibers in the form of self-avoiding chains with appropriate physical and spatial properties (23–25).

In this study, we examined the effects of spatial confinement on chromosome folding using the constrained self-avoiding chromatin (C-SAC) model. We developed a novel algorithm that can generate large ensembles of diverse model chromatin chains in severe spatial confinement, with full excluded volume effect incorporated. We find that spatial confinement is plausibly responsible for much of the observed overall scaling behavior of human chromosome folding. The heterogeneous ensemble of folded model chromatin chains under spatial confinement also predicts chromosome-specific scaling relationships, as well as formation of highly interactive substructures that might give rise to the formation of topological domains. Our findings highlight the importance of nucleus size in regulating the folding landscape of chromosomes.

## MATERIALS AND METHODS

### Model and parameters

**Figure 1.**
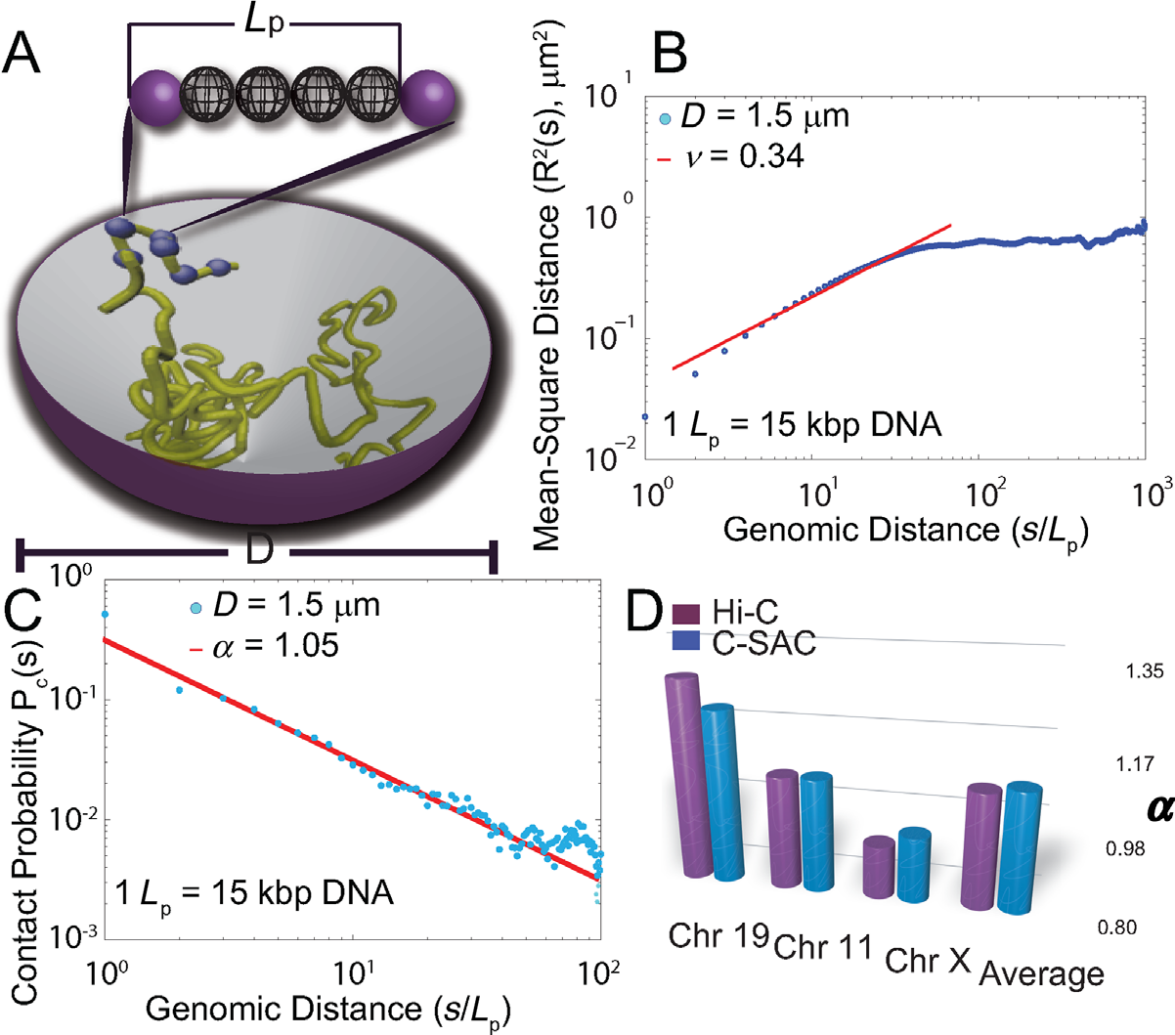
The physical model of C-SAC chains and its scaling properties. (**A**) Schematic representation of the C-SAC model. A chromatin fiber is represented by a self-avoiding polymer chain with a persistence length *L*_p_. Solid spheres are the beads at the boundaries of a persistence unit. Spheres in-between are the interpolated beads inside a unit of *L*_p_. Polymers are grown as chains inside a spherical confined space of a diameter *D*. Beads are not allowed to cross each other or grow beyond the boundary of the spherical volume. (**B**) The scaling of mean-square spatial distance *R*^2^(*s*) from 10 000 chains of length 1000*L*_p_ in log_10_ scale. *R*^2^(*s*) follows a power law of ∼*s*^2*ν*^, with *ν* ∼ 0.34 (95% confidence interval: [0.30, 0.38]), similar to measured *ν* of ∼ 0.33 (6,9). (**C**) The scaling of contact probability *P*_c_(*s*). *P*_c_(*s*) follows a power law of ∼1/*s^α^*, with *α* ∼ 1.05 (95% confidence interval: [1.15, 0.95]), similar to the measured *α* of 1.08 (3). (**D**) Comparison of exponent *α* of contact probability, *P*_c_(*s*), between C-SAC and Hi-C data (3). Values of *α* for different chromosomes from references (3,12) were compared to those calculated separately for different clusters in the C-SAC population of chromatin chains. Black bars denote the experimentally observed exponent α for Chr 19, Chr 11, Chr X and the average *α* across all chromosomes in human genome. Grey bars are αs from the corresponding C-SAC clusters and the average *α* of entire population.

In our C-SAC model, a chromatin fiber is represented as a self-avoiding polymer chain consisting of beads. Each bead has a diameter of 30 nm (26,27) and is 3000 bp long (28,29). Every five beads form a persistence unit, which corresponds to a persistence length of 150 nm (Figure 1A) (29). Our model chain is 4996 beads long, equivalent to about 15 Mbp of DNA.

Each chromatin chain is generated in a confined space of nucleus, which we modeled as a sphere. The sphere diameter, *D*, is selected to be proportional to the size of the human cell nucleus. We assumed an average nucleus size of a diameter of ∼11 μm for 6 billion base pairs of human DNA (1). The diameter of the nucleus for a 15 Mb long chromatin chain is therefore about 1.5 μm. With this model, we grow our chromatin chains sequentially in a sphere of a diameter of *D* = 1.5 μm (Figure 1A). We overcame the difficulties of generating folded chromatin chains inside a small volume by sequentially growing self-avoiding chains one persistence unit at a time using the technique of geometric sequential importance sampling (see Supplementary Information for more details) (23–25,30–33). Subsequently, *D* was changed to *D* = 2.5, *D* = 5.0, *D* = 7.5, *D* = 10.0, *D* = 30.0 and *D* = 500.0 μm to explore the effects of size of confined space on the spatial organization of chromatin.

### Growing chromatin chains using geometric sequential importance sampling

The chromatin chains in 3D space are generated following a chain growth approach (23–25,30–33). A chromatin chain contains *n* persistence units, with the location of the *i*th persistence unit denoted as $x_i=(a_i,b_i,c_i)\in \mathbb {R}^3$. The configuration ***x*** of a full chromatin chain with *n* persistence units is:

\begin{equation*}
{\boldsymbol x}= (x_1, \ldots , x_n).
\end{equation*}

Our target distribution π(***x***) is the uniform distribution of all spatially realizable chromatin chains within the given confinement. To generate a chromatin chain, we grow the chain one persistence unit at a time, ensuring the self avoiding property along the way, namely, *x*_*i*_ ≠ *x*_*j*_ for all *i* ≠ *j*. We use a *k* = 100-state off-lattice discrete model (see (24–25,30–33) for more details). The new persistence unit added to a growing chain with the current persistence unit located at *x*_*t*_ is placed at *x*_*t* + 1_, which is a persistence length *L*_p_ distance away from *x*_*t*_. *x*_*t* + 1_ is randomly taken from one of the unoccupied *k*-sites neighboring *x*_*t*_. As random selection from available empty neighboring sites introduce bias for sampling from π(***x***), we keep track of the bias and assign each successfully generated chain a proper weight *w*(***x***). Details can be found in references (24,30–31).

Each persistence unit further contains [(*L*_p_/*d*_f_) − 1] number of monomer beads, where *L*_p_ = 150 nm, and the fiber diameter *d*_f_ is 30 nm. These monomers are connected by a chain, and their positions are interpolated as if they are on a rigid rod (Figure 1A). This is to mimic the persistence behavior of the chromatin fiber. We again enforce the self-avoiding property, such that these beads will not intersect with any other beads in the partial chain that has already been grown. All together, there are *N*′ = *N* + (*N* − 1)[(*L*_p_/*d*_f_) − 1] = 1000 + 999 [(150/30) − 1] = 4996 monomer beads for a *N* = 1000*L*_p_ unit long chain. For larger confinement space, we generated chains up to *N* = 8100*L*_p_.

### Method validation

#### Scaling of C-SAC chains without confinement

We first used our geometric sequential importance sampling technique to generate free space self-avoiding C-SAC chains without confinement, as their scaling behavior is well understood (34). We generated 10 000 C-SAC chains of different length *N*, for *N* ∈ {100, 200, …, 1000}. The scaling relationship *R*(*N*) ∼ *N^ν^* and *P*_c_ ∼ *N^α^* are shown in Supplementary Figure 1. The scaling exponents are found to be *ν* ∼ 0.59 and *α* ∼ −1.88, which are very close to the expected values of $\nu \sim \frac{3}{5}$ and *α* ∼ −3*ν* (34).

## RESULTS

### C-SAC model gives observed scaling behavior of human chromosomes

We generated ensembles of 10 000 independent self-avoiding model chromatin chains for different chain length *N* of 50, 100, 200 and then up to 1000, with increments of 100 confined to a region of *D* = 1.5 μm. Our C-SAC model chromatin chains exhibit experimentally observed scaling properties. The mean-square spatial distance *R*^2^(*s*) of partial chains of length *s* from 10 000 chains of length *N* = 1000*L*_p_ follows the relationship of *R*^2^(*s*) ∼ *s*^2*ν*^, with an exponent *ν* of ∼0.34 at shorter genomic distances (95% confidence interval of [0.30, 0.38] is obtained by bootstrapping 10 000 chains for 10 000 times), but levels off with *ν* = 0 at larger genomic distances. The experimentally observed *ν* = 0.33 was derived from FISH data between 0.4 Mb and 2.0 Mb (6). In our C-SAC model, *ν* = 0.34 is derived accordingly between 5 and 25*L*_p_, by matching the onset points of the leveling-off effect (10 Mb in the FISH study, and 125*L*_p_ in C-SAC chains) (6) (Figure 1B, see Supplementary Information for additional details and results). Since the mass density of chromatin and how it varies in different loci and different chromosomes are unknown, the regime that the exponents are extracted are not directly comparable by genomic distance to the experimental data. The mass density used in this study is an average property, and it may differ from the actual mass density at the loci measured in the FISH experiments (6). As 125*L*_p_ is where C-SAC chains levels-off and 10 Mb is the genomic length where the leveling-off is observed in FISH experiments (6), we matched 125*L*_p_ to 10 Mb. This allowed us to calculate the exponent *ν* in the same regime of the FISH experiments (6). To characterize the scaling relationship of contact probability *P*_c_(*s*) and contour length *s* between two loci, we harvested partial chains of length *s* from independent ensembles of different full chain lengths and estimated *P*_c_(*s*). As contact probability *P*_c_(*s*) between loci of *s* genomic distance were derived from fragments from different chromosomes in Hi-C studies (3), partial chains from independent ensembles of varying full lengths are necessary to remove self correlations, which may occur when subchains are taken from the same ensemble of chains with a fixed full length as in (3). Our C-SAC model can reproduce the scaling relationship of contact probability *P*_c_(*s*) ∼ 1/*s^α^*, with an exponent *α* of ∼1.05 (Figure 1C), which is in excellent agreement with *α* ∼ 1.08 measured in Hi-C studies (3).

Our C-SAC model also captures observed deviations in *α* from the average in individual chromosomes (12). After clustering the 10 000 C-SAC chains of length *N* = 1000*L*_p_ according to their spatial similarities measured in pairwise bead distances (see Supplementary Information), the resulting 20 clusters have exponent α ranging from 0.79 to 1.3 (Supplementary Table 1). The exponents of these clusters give the full range of *α* observed experimentally. For example, exponents of cluster 10, 15 and 17 agree well with those of Chromosome 19, X and 11/12, respectively (Figure 1D) (3,12). These results are obtained without using any characteristics specific to Chromosome X, 19, 11 or 12. Complex scaling property of human genome arises fully from structural clusters resulting from the spatial confinement. Overall, our results indicate that the restriction of volume imposes strong constraints and chromatin chains under such confinement exhibit experimentally observed scaling behavior of human chromosomes.

### Nuclear size determines chromosomal scaling behavior

**Figure 2.**
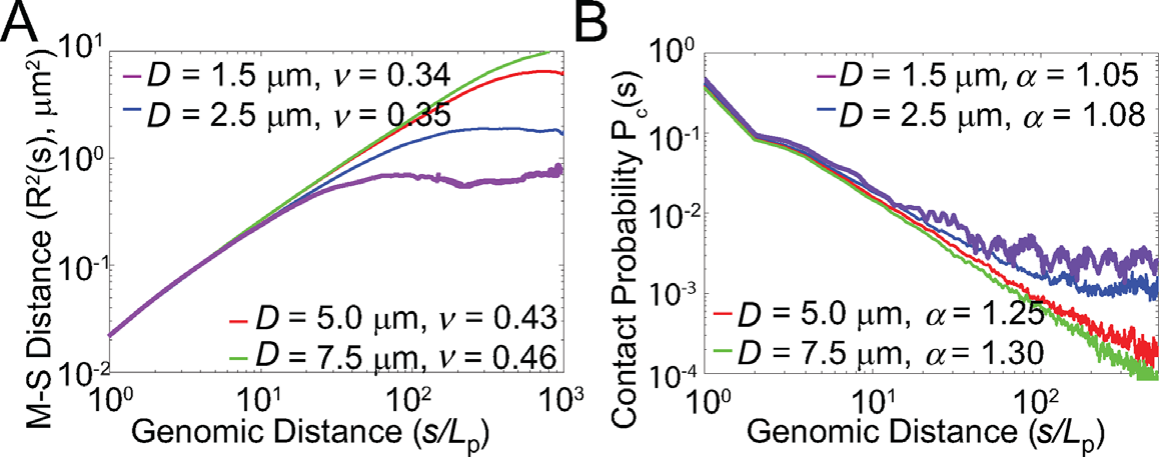
Size of confinement affects the scaling behavior of human chromosomes. (**A**) Mean-square spatial distance *R*^2^(*s*) versus genomic distance *s* for different confinement sizes *D*. For larger nuclei, chromatin chains have larger exponent *ν*. The leveling-off effects disappear as the confinement size increases. (**B**) Contact probability *P*_c_(*s*) versus chain length *s* for different confinement sizes *D*. For larger nuclei, chromatin chains have larger exponent *α*, indicating more open conformations.

To examine the effects of the spatial confinement, we generated independent ensembles of 10 000 C-SAC chains of length *N* inside a sphere *D*. Here *N* is varied from 50, 100 and then up to 1000, with increments of 100. The sphere diameter *D* takes the value of 2.5, 5.0 and 7.5 μm, in addition to 1.5 μm. We independently generated different ensembles of 10 000 C-SAC chains at each of the combination of *N* and *D* values. Altogether, we have 4 × 11 = 44 independent ensembles of 10 000 C-SAC chains for calculating contact probability. We used partial chains of length *s* from the ensemble of 10 000 chains of *N* = 1000 of different *D* for the calculation of mean-square spatial distance *R*^2^(*s*), following the approach used in the FISH studies (6,9). We found that both exponents α and ν increase with *D* (Figure 2A and B). Furthermore, chromatin chains tend to adopt more open conformation as *D* increases. At the same time, the leveling-off effect at larger genomic distances disappears (Figure 2B). Further clustering of chromatin structures (see Supplementary Information) at different nuclear sizes showed that even with the smallest nucleus size of *D* = 1.5 μm, there exists a substantial amount of open chromatin structures (10.9%), while the compact structures and in-between structures are 18.6 and 70.5% of the population, respectively. As the size of the nucleus increases, the percentage of open-like structures in the population increases. These results therefore suggest that nuclear size is a major factor in influencing the overall folding landscape of chromatin, via modulation of the spatial confinement scale *D*.

### Formation of highly interactive substructures upon confinement and topological domains

**Figure 3.**
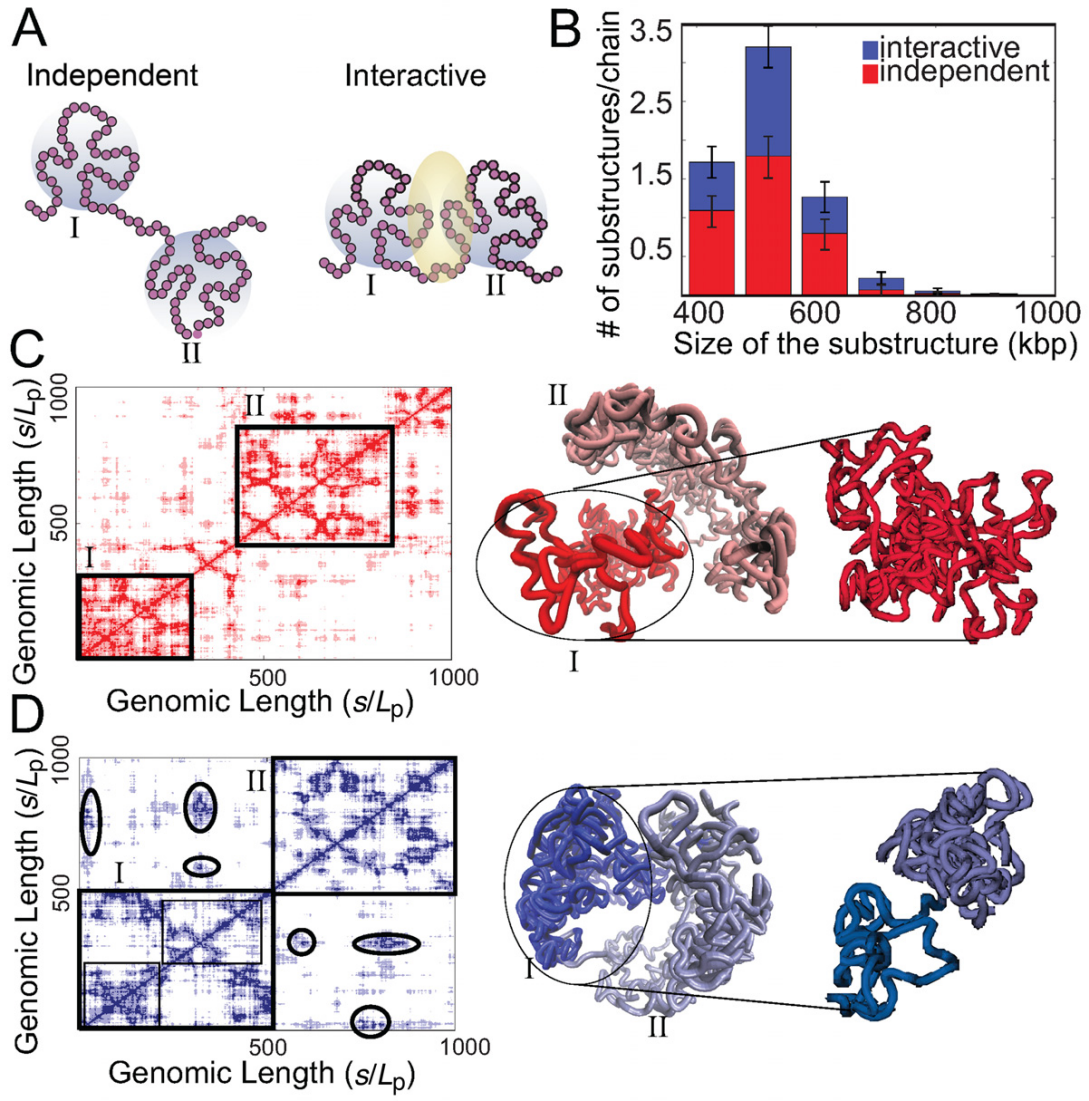
Formation of domain-like substructures upon confinement. (**A**) Illustration of substructures on C-SAC chains, where consecutive monomers are contained in spheres of 800 nm diameter (grey circles). Two independent substructures with no interaction between them, as well as two interactive substructures with >20% of their monomers participating interaction (interface shaded) are shown. (**B**) The distribution of number of substructures per chain containing different amount of DNA for both independent and interactive categories are shown. (**C**) A random C-SAC chromatin chain with independent substructures. The rotated and zoomed-in substructure shows a singular domain-like conformation. The domain-like substructures can also be seen in the corresponding distance matrices, where the spatial distances between different loci of the C-SAC model chains are color coded, with darker shade representing interactions between chromatin beads. The chromatin chain contains two highly interactive substructures that do not interact with each other. (**D**) A random C-SAC chromatin chain with substructures are shown. There are two small interactive substructures, as can be seen in the rotated and zoomed-in conformation. The domain-like substructures can also be seen in the corresponding distance matrices, where the spatial distances between different loci of the C-SAC model chains are color coded, with darker shade representing interactions between chromatin beads. The chromatin chain contains two highly interactive substructures that interact with each other. Circles highlight regions of interactions.

We used the C-SAC model to further explore structural properties of chromatin fibers. Topological domains were previously observed in electron microscopy studies (11,35–36) and in recent 3C-based studies (Hi-C) (37,38). Such domains are distinctive regions along the chromatin chain with significantly elevated interactions within region (37). Their DNA content range from a few kbp to 1 Mbp, and they occupy a volume of 300–800 nm in diameter (39). To examine whether C-SAC chains contain domain-like substructures, we calculated the number of consecutive persistence units in spheres of 800 nm diameter along the chromatin chain. If a sphere contains chromatin fragments that contain more than 400 kbp DNA, it is regarded as a highly interactive substructure. We further define two types of substructures: (i) interactive substructures in which more than 20% of their persistence units are in spatial proximity with those of other interactive substructures, and (ii) independent substructures in which none of their units are in spatial proximity with any units of other substructures (Figure 3A).

On average, there are ∼6.5 substructures per chain, which occupies around 21% of the entire 15 Mbp C-SAC chain. 41% of these substructures are interactive, whereas the rest of them are independent substructures (Figure 3B). Existence of these highly interactive substructures are also observed from interaction matrices and 3D conformations of individual chains (Figure 3C and D).

In summary, there exists distinct substructures in C-SAC chromatin chains with elevated interactions. These results are observed without requiring special simulation conditions or specific binding sites as in other chromatin models (3,12,16). Their existence suggests that the confinement of the cell nucleus is sufficient to induce tentative formation of highly interactive substructures along the chromatin chains, which could further give rise to the formation of topological domains.

### Scaling behavior of human chromosomes is not altered by random binder-mediated looping interactions

Several polymer models of long-range chromatin organization are based on the introduction of explicit looping probability or looping through binder-mediated interactions (12,17). To assess how chromatin looping in addition to confinement would affect the scaling behavior of chromatin chains, we distribute different numbers of binding sites randomly along the chromatin chains, which cover from 10 to 50% of the total number of persistence units in the chromatin fiber. Chromatin structures with a large number of binding sites in spatial proximity are subject to binder-mediated interactions. These structures will then have lower energy and therefore higher probability of presence in the chromatin population. We calculated the distribution of the chromatin chains with such binder interactions, in which the binding energy of connecting two interacting sites is assigned to be 6*k*_B_*T* (12,40) (see Supplementary Information). This allows us to assess the scaling properties of different populations of chromatin chains under different looping conditions.

**Figure 4.**
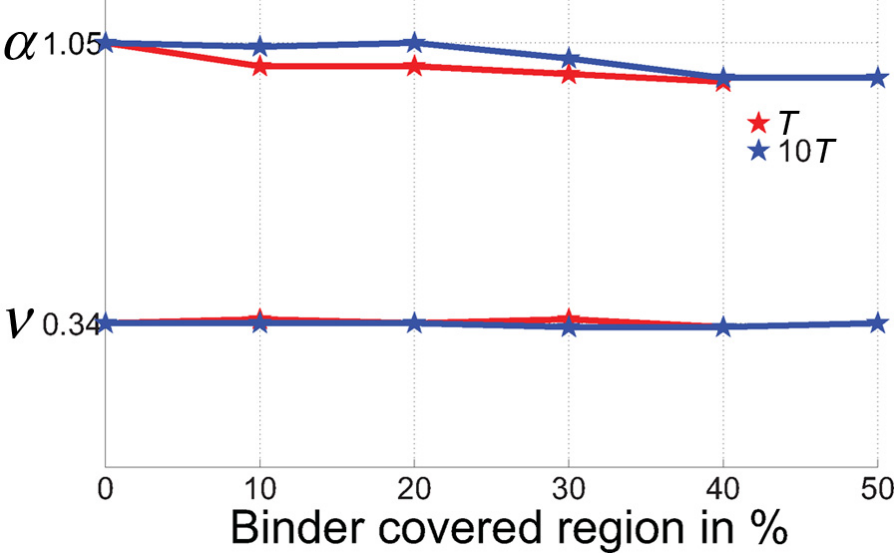
Scaling exponents α and ν when different fractions of C-SAC chromatin chains are covered by binding sites. The binding energy is assigned to 6*k*_*B*_*T* (12) for two relative temperatures (*T* in grey, 10*T* in black). Neither scaling exponents experiences significant changes as the binder coverage increases.

Our results showed that there is virtually no change in the scaling exponents *α* and *ν* in C-SAC chains after introducing binders compared to the original C-SAC chains, where the only constraint is the spatial confinement of the cell nucleus (Figure 4). These results indicate that random self-avoiding chromatin chains folded inside a confined space have an intrinsic propensity to form loops, without the explicit introduction of additional binders. Overall, our results indicate that the confinement at the scale *D* is the dominant factor in determining the average scaling behavior of chromatin structures.

### Relevant size regime of chromatin confinement for the spatial organization of chromosomes

Chromosomes are found to occupy localized territories of the size of ∼2 μm diameter (11). To span a chromosome territory, only a short fragment of chromatin fiber (*ca.* 150 kb, assuming 30 nm diameter and 150 nm persistence length) is required, which is well below the range of 0.5–7.0 Mb measured in Hi-C studies (3). Studying the scaling behavior of the self-avoiding polymer chains in the correct confinement is therefore the key to construct the relevant model for understanding chromosome folding. We used C-SAC model to explore the relationship between the size of confinement and the scaling properties of confined chromatin chains, as the calculated scaling exponents of C-SAC chains in the relevant size regime (*α* ∼ 1.05) is well below the theoretical scaling exponent of self-avoiding polymer chains in confinement (*α* ∼ 1.50) (34).

**Figure 5.**
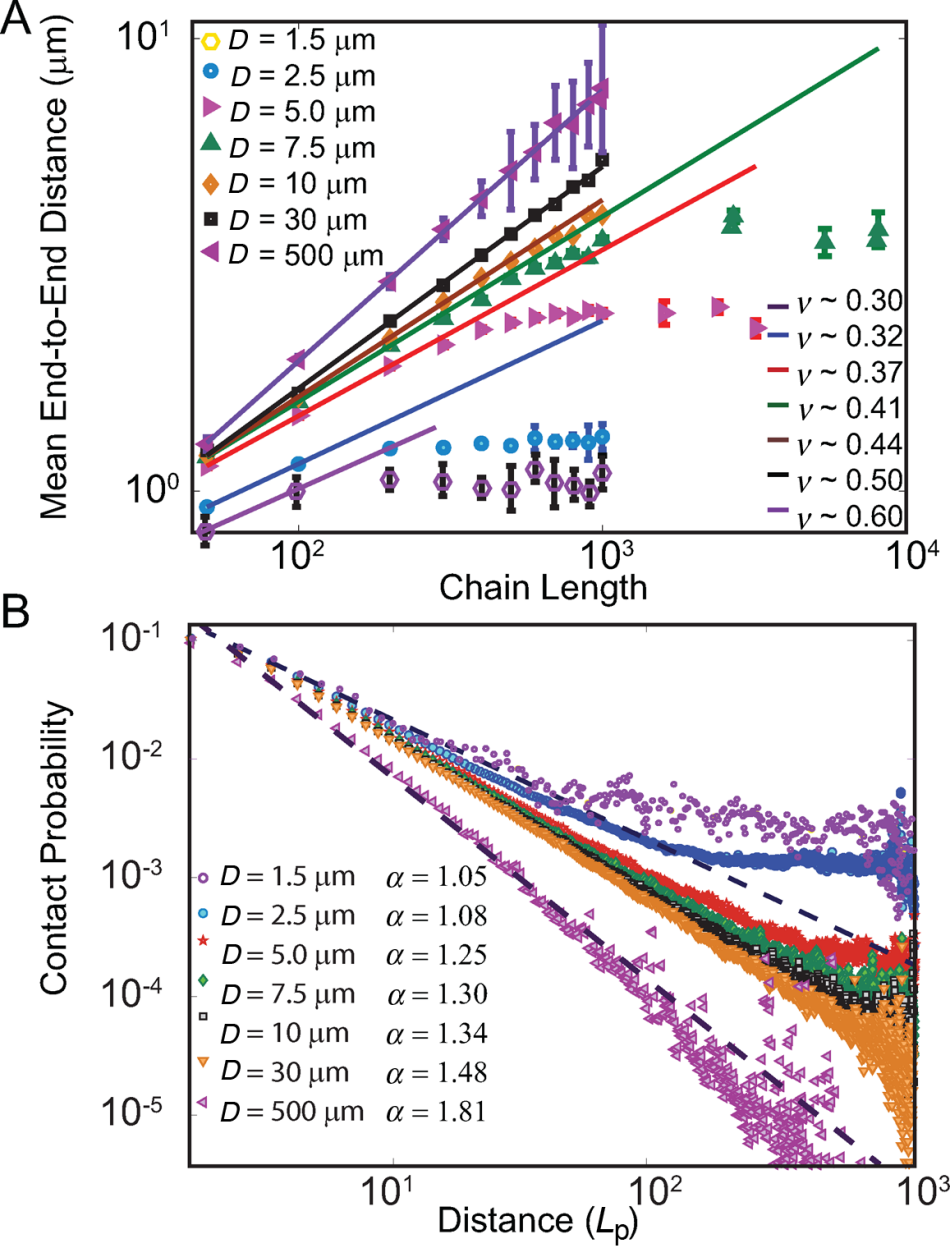
Scaling properties of self-avoiding C-SAC chains in confinement. (**A**) Relationship between the mean end-to-end distance and the chain length. Each data point is an average of 10 000 chains of different length under specific confinement of diameter *D*. As *D* increases, the scaling behavior of self-avoiding walks converges to that of ideal SAW (*ν* = 0.6). (**B**) Relationship between mean contact probability and partial chain length *s*. Each data point is an average of 10 000 chains of different length *N* under different confinement of diameter *D*.

We generated independent ensembles of C-SAC chains in equilibrium and calculated the relationship between the chain length and the end-to-end distance, as well as the relationship between contact distance and the contact probability. In total, we generated chains with different length *N* of 50 , 100 , …, 1000 at increment of 100 each with five different confinements *D* (*D* = 1.5, 2.5, 10, 30 and 500 μm). We also generated C-SAC chains of length *N* from 50 to 8100 at different increments for two different confinements *D* (*D* = 5.0 and 7.5 μm) (Figure 5). We found that chromatin chains exhibit confinement-dependent scaling behavior, with *ν* ranging from 0.30 to 0.60 (Figure 5A). That is, the mean end-to-end distance of self-avoiding C-SAC chains in a spherical confinement is a function of both the chain length *N* and the confinement diameter *D*, when the length of *N* is larger than *D* (see Supplementary Figure S2). This confinement-dependent regime is illustrated in Figure 5 for both mean end-to-end distance and contact probability. Figure 5B includes data presented in Figure 2B, with additional data for *D* of 10, 30 and 100 μm to depict comprehensively the relationship between α and the genomic distance. This helps to illustrate the important issue of the cross-over regime for self-avoiding chromatin and the convergence of the scaling exponent *α*. The asymptotic relationship of *α* = 3*ν* (34) is well-satisfied at larger *D* value, but less so at smaller *D*, as the leveling-off effects take place at shorter chain lengths with more severe confinement at smaller *D*.

Severe spatial confinement has pronounced effects on the conformations of self-avoiding polymers. Overall, we find that the effective scaling exponent slowly changes with increasing *D*, reflecting a rather slow convergence to the asymptotic behavior expected from simple polymer scaling theory (34).

## DISCUSSION

Chromosomes reside within the severely confined space of the cell nucleus. However, the direct effects of nuclear confinement on chromatin folding and compaction are unknown. A major challenge is the extreme difficulty in adequate sampling of long self-avoiding chromatin chains in the confinement of the cell nucleus. The C-SAC model and the novel sampling technique developed in this study enabled us to generate a large number of chromatin conformations in confinement.

Our results showed that the spatial confinement of ∼15 Mb chromatin within regions of diameter *D* of 1.5 μm gives rise to the chromosomal scaling relationships of the average *α* ∼ 1.05 (3) and the average *ν* ∼ 0.34, as well as the leveling-off effects observed experimentally (6,9). Our model also captured the complex folding behavior of the chromosome-specific variation in scaling (12). In addition, the tentative formation of domains (11,35–38) also emerged in C-SAC model as highly interactive substructures, without the need of introducing additional binder molecules and fine tuning of their concentrations. These interactive substructures could be stabilized by introducing more specific interactions through evolutionary selection pressure to form functional topological domains.

We found that *D*, and therefore nuclear size, is a major factor in influencing the overall folding landscape of chromatin. As nuclear size changes, there are significant differences in the chromosome architecture, which are reflected in variations in the scaling exponents. These conclusions are in good agreement with results from Hi-C studies using different cell lines (3,12,18,37). For example, lines of differentiated cells (GM06990 (3), GM12878 (18), IMR90 (37)) have similar overall average scaling behavior, with *α* ∼ 1.08 (3,12) while embryonic stem cells (hESC) (37) behave differently, with an *α* close to 1.6 (12). A characteristic property of an hESC nucleus is that it occupies almost the entire cell volume (41,42) and is plastic and deformable (43). This provides an enlarged space for chromosome organization. As a result, hESC chromatin is largely diffuse (41). Our calculation also showed an increase in *α* when the confined space is enlarged (Figure 2B). This observed variation in scaling corresponds well with the confinement of different nuclear sizes.

The average compactness of the chromatin chains and the fractions of open, compact and in-between chromatin structures are all different when the nuclear size is changed. Nuclear size likely alters the overall structural organization of chromosomes, allowing previously unlikely long-range interactions to occur, at the same time prohibiting certain other genomic interactions present at a different nucleus size. Thus, nuclear confinement may bias distant sites towards spatial proximity.

Our results showed that randomly placed binders do not directly affect the scaling behavior. Biological binders such as CTCF may play more specific roles of modifying or biasing chromosomes toward formation of specific domains required for cell function^44–46^). Future work on the selection of properly placed CTCF binding and its effects will likely be fruitful for understanding the effects of biochemical binding on spatial organization of chromosome.

We compared predictions from C-SAC models with those from other chromatin models. As experimentally observed *ν* ∼ 0.3 deviates significantly from the expected *ν* of 0.5 for sub-chains in equilibrium globule, chromatin fibers were conjectured to be in non-equilibrium fractal globule (FG) state, in which the exponent of *ν* ∼ 0.3 would be retained at every scale (3,16). The lack of leveling-off effects in *P*_c_(*s*) with *s* observed in (3) is consistent with the prediction of the FG model. However, leveling-off effects are observed in FISH studies on different chromosomes at several different length scales (6,9). These leveling-off effects are not accounted for by the FG model. In addition, the significant variation of exponent α among different chromosomes of human cells (3,12) is not explained by the FG model.

An important consideration in studying the scaling relationship of chromosomes is the relevant size regime dictated by experimental observations. An average of 50–100 Mbp chromosome occupies a territory of size ∼2 μm (11). As a result, a chromatin must traverse back and forth many times in the chromosome territory, and severe spatial confinement is at play and will have pronounced effects on the folding and scaling of chromatin fibers. General asymptotic scaling analysis of polymers is overly simplistic to offer much insight under such strong effects of finite sizes. Conventional simulation studies based on Metropolis Monte Carlo are also challenged to generate adequate samples to study the equilibrium ensemble of severely confined chromatin chains in the relevant regime.

Simulation using the novel technique of geometric sequential importance sampling allows the effects of finite size of confinement to be examined in detail. Our results offer an alternative explanation on the scaling relationship of chromosomes to the existing FG (3,16) and SBS (12) models. Overall, our results show that equilibrium ensemble of C-SAC chromatin chains under severe confinement of scale *D* = 1.5 μm exhibit scaling behavior consistent with known experimental data, which are different from that of asymptotic random chains in the relevant biological scale.

A useful result that can be inferred from our analysis is that chromosomes are restricted via confinement of sub-chromosomal regions of size about 15 Mb, each within a *D* of about 1.5 μm-diameter region. Therefore, it may be useful to consider the nucleus to be made up of close-packed regions of size *D*, each containing ∼15 Mb of DNA. For example, one can consider whole chromosomes to be made up of individual units of 15 Mb of DNA, confined to spherical regions of diameter ∼1.5 μm. The whole human nucleus containing ∼6 Gbp of DNA can be considered to be a collection of ∼6000/15 = 400 such units, which can be fit into a nucleus of diameter ∼400^1/3^*D* ≈ 7.4*D* ∼ 11 μm, compatible with the observed size of human cell nuclei (1) (see Supplementary Information). Therefore, the subchromosome confinement parameter *D*, namely, the size of a region containing 15 Mb of DNA, is an important parameter in our structural model.

As spatial confinement is a dominant factor in determining chromosome folding, the specific epigenetic state of genes and transcription activities in different cell types are likely to be influenced by the degree of nuclear confinement. Cell nucleus size at different developmental stages or physiological states may be altered to induce different chromosome folding landscape, enabling different genetic programming to be activated. Overall, how nuclear size and shape relate to cell size and shape, and how their relative ratio or pattern regulate the epigenetic programs of the cells at different developmental stages are important problems requiring further investigations.

Although our approach can generate a large ensemble of chromatin chains under spatial confinement, there still exists uncertainty in the physical parameters used in the current C-SAC model, including the persistence length, the chromatin fiber diameter and the mass density (47). In addition, current chromatin models are based on growing a single chromosome chain, and cannot be used to study inter-chromosomal interactions. Another question is how the 15 Mb sequence scale and the parameter *D* are controlled in the cell. These issues will likely be resolved when chromosomal properties are better understood and the C-SAC algorithm is further improved. It is interesting that our simplistic model can capture complex folding characteristic of human genome. The current study highlighted the importance of spatial confinement in dictating the chromatin folding landscape. With the accumulation of high resolution chromosome conformation capture data, it is envisioned that more specific spatial information inferred from 3C-based studies can be incorporated into the C-SAC model, and realistic ensemble of chromatin conformations reflecting 3C-based information can be reconstructed to gain insight into the structural basis of gene regulation and expression.

## SUPPLEMENTARY DATA

Supplementary Data are available at NAR Online.

SUPPLEMENTARY DATA
