# Supplementary material for: Spatial confinement is a major determinant of the folding landscape of human chromosomes
Source: Nucleic Acids Res. 2014 Jul 2;42(13):8223–30. doi: 10.1093/nar/gku462 (PMC4117743; doi:10.1093/nar/gku462)
Supplement: SUPPLEMENTARY DATA [file supp_42_13_8223__index.html]

Spatial confinement is a major determinant of the folding landscape of human chromosomes — SUPPLEMENTARY DATA 

# Spatial confinement is a major determinant of the folding landscape of human chromosomes

## SUPPLEMENTARY DATA

**Files in this Data Supplement:**

- SUPPLEMENTARY DATA
