# Supplementary material for: Spatial confinement is a major determinant of the folding landscape of human chromosomes
Source: Nucleic Acids Res. 2014 Jul 2;42(13):8223–30. doi: 10.1093/nar/gku462 (PMC4117743; doi:10.1093/nar/gku462)

1 Supplementary Information

1.1 SI Text

1.1.1 Materials and Methods:

Resampling. To improve the success rate of generating full length chromatin chains, we employ the technique of resampling [1, 2, 3, 4, 5, 6, 7]. When there is no unoccupied neighboring sites inside the confined space for x_t of a partially grown chain, there is no place to grow x_{t+1} the next persistence unit, and the chain runs into a dead end. In this case, we go back one step and re-grow the chain at x_{t-1} from the $(t-1)^{th}$ monomer. There are also chains with small weights due to biased sampling. They contribute little to the estimation of properties of the population of chromatin chains.

We employ a simple resampling scheme to address these issues. At each t-th step of the chain growth process, we sort all chains by their weights, and divide them into k fractions. The fraction of chains with the lowest weights are then replaced by copies of chains in the fraction of the highest weights. Weights of these chains are then adjusted accordingly. We then continue to grow chains of this new population. This is repeated until all chains reach full length. We use t=10 and k=3. Details of the resampling strategies can be found in references [1, 2, 3, 4, 5, 6, 7].

We also employ a dynamic resampling scheme [1] when the chain length N is large and/or the sphere diameter D is small. At each step of chain growth process, we calculated effective sample size [1] as:

$$ESS = \frac{\left(\sum_{j=1}^{M} w_i\right)^2}{\sum_{j=1}^{M} w_i^2},$$

where M is the total number of chains. If $ESS < 0.3M$, we assign a probability $p(i)$ to each partial chain i as $p(i) = \exp(w_i - \max_{1\le i\le M} w_i)$ and sample M chains with replacement according to $p(i)$ and adjust the weights of each selected chain k as $w_k^* = \frac{w_k}{p(k)}$. We then continue to grow chains of this new population. This is repeated until all chains reach full length.

Chromatin properties. With m successfully generated chromatin chains, we can calculate the physical properties of the population of chromatin fibers. Denote the configurations of the j-th successfully generated chromatin chain as $\boldsymbol{x}^{(j)} = (x_1^{(j)}, \cdots, x_n^{(j)})$, and its associated weight $w^{(j)}$. To calculate the mean value of a

physical property $\bar{h}(\boldsymbol{x})$ such as the mean end-to-end distance of a chromatin chain, we have:

$$\bar{h}(\boldsymbol{x}) = \mathbb{E}_{\pi(\boldsymbol{x})}[h(\boldsymbol{x})] = \frac{\sum_{j=1}^{m} h(\boldsymbol{x}^{(j)}) \cdot w^{(j)}}{\sum_{j=1}^{m} w^{(j)}}.$$

Mean end-to-end distance. The mean end-to-end distance $R(N)$ is the mean Euclidean distance between the beginning and the end of the chain of a length N. For the jth chromatin chain, we have:

$$R(N)^{(j)} = \|x_1^{(j)} - x_N^{(j)}\|.$$

The mean end-to-end distance is then calculated for the set of m chromatin chains as:

$$\bar{R}(N) = \frac{\sum_{j=1}^{m} R_{(j)}(N) \cdot w^{(j)}}{\sum_{j=1}^{m} w^{(j)}}.$$

Mean-square spatial distance. The mean-square spatial distance $R^2(s)$ is the mean-square Euclidean distance between genomic regions with a genomic separation s, here in units of persistence length. For the jth chromatin chain, we have:

$$R^2(s)^{(j)} = \frac{\sum_{i=1,\, j=i+s} \|x_i^{(j)} - x_j^{(j)}\|^2}{N - s},$$

where the denominator $N - s$ is the total number of all possible such interactions with s-separations. The mean-square spatial distance is then calculated for the set of m chromatin chains as:

$$\bar{R}^2(s) = \frac{\sum_{j=1}^{m} R^2_{(j)}(s) \cdot w^{(j)}}{\sum_{j=1}^{m} w^{(j)}}.$$

Contact probability. The contact probability $P_c(s)$ is the probability of two genomic regions separated by genomic distance s to be in spatial proximity of each other for chain of length N. Following Lieberman-Aiden et al. [8], it is calculated by counting the number of times that the Euclidean distance between two regions separated by genomic distance s is smaller than a distance threshold d_θ, divided by the number of all such candidate contacts. Let $\mathbb{I}_c^{(k)}(s)$ be the observed number of i and j contacts that satisfies the condition $\|x_i - x_j\| \leq d_\theta$, with $j - i = s$ in chain k. Let $\mathbb{I}_{\text{all}}(s)$ be the number of all possible contacts of two regions

separated by a genomic length of s and is equal to $N - s$. An estimate of contact probability $P_c^{(k)}(s)$ for chain k of length N is:

$$\hat{P}_c^{(k)}(s) = \frac{\mathbb{I}_c^{(k)}(s)}{\mathbb{I}_{\text{all}}(s)}.$$

The mean value from the weighted ensemble average is then calculated as:

$$\bar{P}_c(s) = \frac{\sum_k P_c^{(k)}(s) \cdot w^{(k)}}{\sum_k w^{(k)}}.$$

Reweighting. As chromatin chains are generated following the uniform distribution $\pi(\boldsymbol{x})$ of all geometrically realizable chains, these samples need to be reweighted in order to calculate ensemble properties of chromatin chains following a different distribution $\pi'(\boldsymbol{x})$.

To asses the effect of specific binding on the population of chromatin chains, we recalculate the associated weights of each chain for chromatin following the new distribution $\pi'(\boldsymbol{x})$, which is the Boltzmann distribution after incorporating energies of binding interactions. For a chromatin chain with interactions mediated through protein binder, each interaction between any (i, j) pairs of sites contributes to the weight of the chain by the Boltzmann factor of $\exp(E^{(k)}(i, j)/k_B T)$. Here $E^{(k)}(i, j)$ is the binding energy if both i and j contain binding sites and are mediated by the binder protein, otherwise $E^{(k)}(i, j) = 0$. The total weight of the kth chain previously sampled from the uniform distribution is then re-calculated as:

$$w^{(k)} = \prod_{(i, j)} \exp(E^{(k)}(i, j)/k_B T).$$

Clustering. We clustered the generated chromatin chain conformations according to their pairwise distances between persistence units using a k-means clustering algorithm [9]. For k-means clustering, we need to calculate the Euclidean distances between persistence units. As we have a population of m=10,000 chains, each with $N = 1,000$ persistence units, this amounts to $n = N \times (N - 1)/2 = 499,500$ number of pairwise distances to be calculated. Since the algorithm is of $O(m^{nk+1} \log m)$-complexity, we coarse-grained each chain to speed up the computation. We take sequentially every 33 persistence units as our new unit. This gives 30 connected units, where the number of pairwise distances is now $n = 30 \times 29/2 = 435$. We set the number of clusters k to 20.

1.1.2 Additional Details of Results

Scaling of C-SAC chains without confinement. We first used our geometric sequential importance sampling technique to generate free space self-avoiding C-SAC chains without confinement. The scaling relationship $R(N) \sim N^{\nu}$ and $P_c \sim N^{\alpha}$ are shown in SI Fig. 1.

Scaling of confined C-SAC chains. In a spherical confinement, the mean end-to-end distance of polymer chains is a function of both the chain length N and the confinement diameter D, if the length of N is larger than D. This confinement dependent regime is illustrated in SI Fig. 2, where $R(N)$ measured from independent ensembles of 10,000 C-SAC chains of different length N under different confinement D are plotted against N/D^3.

α fit is obtained from the region before contact probability become noisy (from 3 to 40 L_ps).

$R(s) \sim s^{\nu}$ scaling relationship from FISH studies. In the FISH study of ref. [11], spatial distances between different loci with different genomic separation s were measured on two different subchromosomal regions of Chr 12 of mouse pre-pro-B cells and pro-B cells. Our non-linear fit (SI Fig. 3A-B and Fig. 3C-D) gives a scaling exponent of $\nu \sim 0.37$ when $s < 0.5$ Mb for pre-pro-B cells, and $\nu \sim 0.27$ when $s < 0.5$ Mb for pro-B cells. The leveling-off effects takes place at $s = 0.5$ Mb in mouse Chr 12 of both pre-pro-B cells and pro-B cells.

In the FISH study of human Chr 11 and Chr 1 [12], ν was reported to be ~ 0.33 in both human Chr 11 Chr 1 when $0.4 < s < 2$ Mb. The leveling-off effects were reported to takes place at $s \geq 10$ Mb in Chr 11 and $s \geq 3$ Mb in Chr 1 [12].

It was also reported in ref. [13] that the FISH study of mouse Chr 14 in ref. [14] exhibits a $\nu \sim 0.5$ when $s < 3.5$ Mb, beyond which the leveling-off effects may take place.

$R(s) \sim s^{\nu}$ scaling of confined C-SAC chains and comparison with FISH studies. In C-SAC chains of length $N = 1,000$ with the confinement of $D = 1.5$ μm, the leveling-off effects are found to take place at around $s = 125L_p$. We calculated the scaling exponent ν of $R(s) \sim s^{\nu}$ between $s = 5L_p$ and $s = 25L_p$. This choice of $25L_p$ is based on the ratio of 25/125, which is the same as the ratio of 2Mb/10Mb between the distance threshold where ν was fitted and the distance threshold beyond which the leveling -off effects occurred in human Chr. 11 [12], which was also used in the study of refs. [8, 15]. We found $\nu \sim 0.34$ when $5L_p \leq s \leq 25L_p$.

As discussed above, there are some variations in the reported values of the scaling exponent ν from existing FISH studies. Similarly, we found that ν also varies depending on the regime where the exponents were fitted. If $s \leq 60L_p$, ν is found to be ~ 0.25, and $\nu \sim 0.5$ if $s \leq 15L_p$.

Details of scaling of α and ν of each of the $k = 20$ clusters. The average scaling exponents α and ν of each cluster, along with the size of the cluster are listed in Table S1.

References

[1] Liu,J.S., and Chen,R. (1998) Sequential monte carlo methods for dynamic systems. *Journal of the American Statistical Association* **93**(443):1032-1044.

[2] Liang,J., Zhang,J., and Chen,R. (2002) Statistical geometry of packing defects of lattice chain polymer from enumeration and sequential Monte Carlo method. *The Journal of Chemical Physics* **117**:3511–3521.

[3] Zhang,J., Chen,R., Tang,C., and Liang,J. (2003) Origin of scaling behavior of protein packing density: A sequential monte carlo study of compact long chain polymers. *The Journal of Chemical Physics* **118**:6102.

[4] Lin,M., Lu,H.M., Chen,R., and Liang,J. (2008) Generating properly weighted ensemble of conformations of proteins from sparse or indirect distance constraints. *The Journal of chemical physics* **129**(9):094101.

[5] Lin,M., Chen,R., and Liang,J. (2008) Statistical geometry of lattice chain polymers with voids of defined shapes: sampling with strong constraints. *The Journal of Chemical Physics* **128**(8):084903.

[6] Zhang,J., Dundas,J., Lin,M., Chen,R., Wang,W., and Liang,J. (2009)Prediction of geometrically feasible three dimensional structures of pseudoknotted RNA through free energy estimation. *RNA* **15**: 2248–2263

[7] Lin,M., Zhang,J., Lu,H.M., Chen,R., and Liang,J. (2010) Constrained proper sampling of conformations of transition state ensemble of protein folding. *The Journal of chemical physics* **134**(7):075103.

[8] Lieberman-Aiden,E., van Berkum,N.L., Williams,L., Imakaev,M., Ragoczy,T., Telling,A., Amit,I., Lajoie,B.R., Sabo,P.J., Dorschner,M.O., Sandstrom,R., Bernstein,B., Bender,M.A., Groudine,M.,

Gnirke,A., Stamatoyannopoulos,J., Mirny,L.A., Lander,E.S., and Dekker,J. (2009) Comprehensive mapping of long-range interactions reveals folding principles of the human genome. *Science* **326**:289-293.

[9] Hartigan,J.A., and Wong,M.A. (1976) Algorithm AS 136: A k-means clustering algorithm. *Journal of the Royal Statistical Society* **28**(1):100-108.

[10] de Gennnes,P.G. (1979) in *Scaling Concepts in Polymer Physics.* (Cornell University Press, New York) *pp* 1–100.

[11] Jhunjhunwala,S., van Zelm,M.C., Peak,M.M., Cutchin,S., Riblet,R., van Dongen,J.J., Grosveld,F.G., Knoch,T.A., and Murre,C. (2008) The 3D structure of the immunoglobulin heavy-chain locus: implications for long-range genomic interactions. *Cell* **133**:265-279.

[12] Mateos-Langerak,J., Bohn,M., de Leeuw,W., Giromus,O., Manders,E.M., Verschure,P.J., Indemans,M.H., Gierman,H.J., Heermann,D.W., van Driel,R., and Goetze,S. (2009) Spatially confined folding of chromatin in the interphase nucleus. *Proceedings of the National Academy of Sciences of the United States of America* **106**:3812-3817.

[13] Barbieri,M., Chotalia,M., Fraser,J., Lavitas,L.M., Dostie,J., Pombo,A., and Nicodemi,M. (2012) Complexity of chromatin folding is captured by the strings and binders switch model. *Proceedings of the National Academy of Sciences of the United States of America* **109**:16173-16178.

[14] Langmead,B., Trapnell,C., Pop,M., and Salzberg,S.L. (2009) Ultrafast and memory-efficient alignment of short DNA sequences to the human genome. *Genome Biol.* **10**(3), R25.

[15] Mirny,L.A. (2011) The fractal globule as a model of chromatin architecture in the cell. *Chromosome Research* **19**:37-51.

Table 1: Scaling Exponents of the 20 Clusters of Chromatin Chains

Cluster	Size	α	ν
1	458	0.99	0.37
2	122	NA	NA
3	70	NA	NA
4	816	1.02	0.37
5	806	1.13	0.37
6	436	1.13	0.38
7	232	0.87	0.36
8	86	NA	NA
9	560	1.17	0.37
10	560	0.96	0.37
11	666	1.16	0.37
12	388	1.14	0.37
13	496	1.05	0.38
14	332	0.79	0.37
15	374	1.08	0.38
16	1078	1.14	0.37
17	534	1.28	0.37
18	882	1.03	0.37
19	284	1.12	0.40
20	820	1.14	0.38

Figure 1: Scaling properties of C-SAC chains in free space. **(A)** $R(N)$ vs. N relationship in log-scale. Each data point is derived from an ensemble of 10,000 chains of length N. The exponent ν is found to be 0.59, similar to the theoretical exponent for three-dimensional SAWs in good solvent [10]. **(B)** P_c vs. N relationship in log-scale. The scaling expoent α is approximately -1.88, similar to the theoretical exponent of $-3\nu = -1.8$ for three-dimensional SAWs in good solvent [10].

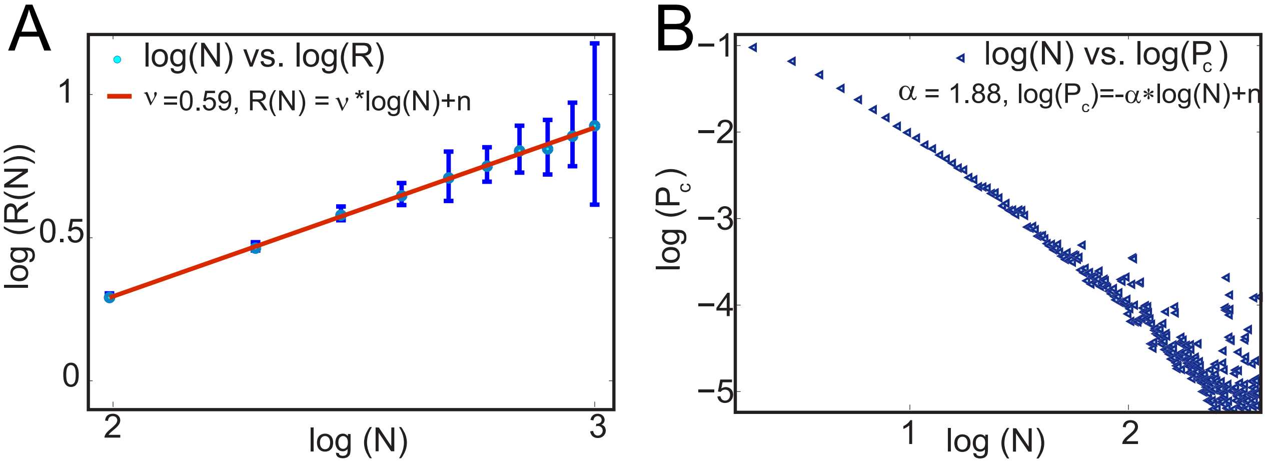

Figure 2: Scaling properties of C-SAC chains in confinement. **(A)** Relationship between $R(N)$ and N/D^3 of C-SAC chains in confinement. Each data point is an average of 10,000 chains of length N under a specific confinement of D. Under severe confinement when D is small, $R(N)$ is influenced not only by the length of the polymer but also the size D of the confinement. **(B)** Relationship between $R(N)$ and N of C-SAC chains in confinement. Each data point is an average of 10,000 chains of length N under a specific confinement of D. As D increases, the scaling behavior of chromatin chains converges to that of ideal SAWs.

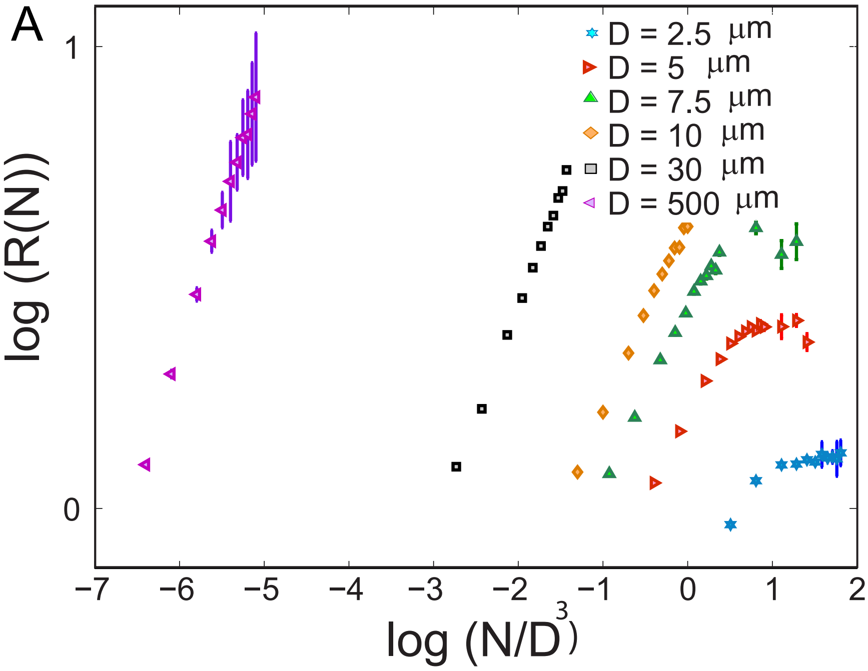

Figure 3: Mean spatial distance $R(s)$ of two different subchromosomal regions. **(A)** The scaling behavior of $R(s)$ of FISH data spanning 3 Mb when the probe is anchored in genomic element BAC [11] (red triangle) in pre-pro-B cells chromosome 12 of mouse genome. **(B)** The scaling behavior of $R(s)$ of FISH data when the probe is anchored in genomic element h1 [11] (red triangle) in pre-pro-B cells chromosome 12 of mouse genome. **(C)** The scaling behavior of $R(s)$ of FISH data when the probe is anchored in genomic element BAC [11] (red triangle) in pro-B cells chromosome 12 of mouse genome. **(D)** The scaling behavior of $R(s)$ of FISH data when the probe is anchored in genomic element h1 [11] (red triangle) in pro-B cells chromosome 12 of mouse genome.

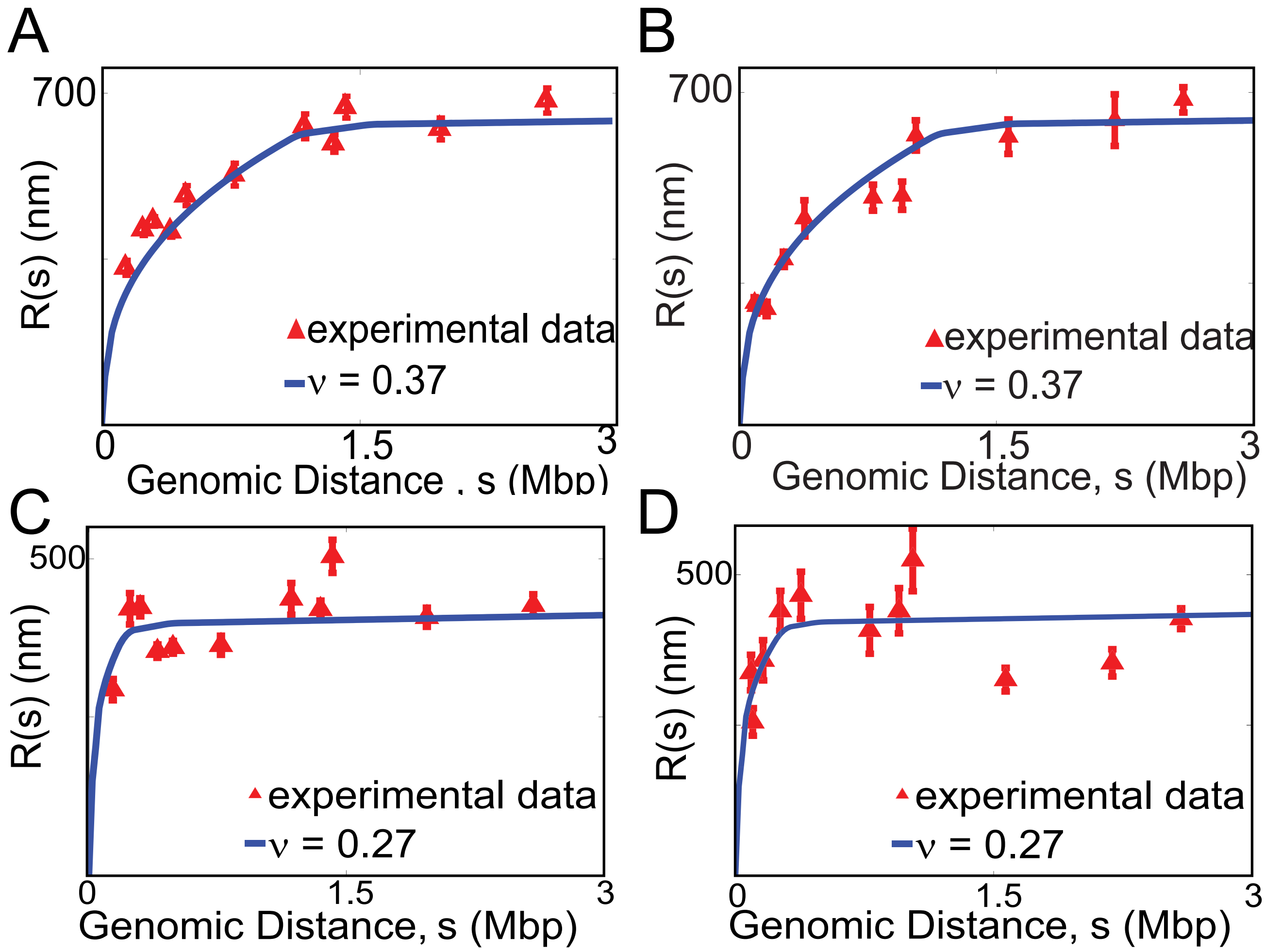

Supplement: SUPPLEMENTARY DATA [file supp_gku462_nar-00876-n-2014-File008.pdf]
